# Inadequately Treated Wastewater as a Source of Human Enteric Viruses in the Environment

**DOI:** 10.3390/ijerph7062620

**Published:** 2010-06-14

**Authors:** Anthony I. Okoh, Thulani Sibanda, Siyabulela S. Gusha

**Affiliations:** Applied and Environmental Microbiology Research Group (AEMREG), Department of Biochemistry and Microbiology, University of Fort Hare, P/Bag X1314, Alice 5700, South Africa; E-Mails: sgusha@ufh.ac.za (S.S.G.); sibandath@yahoo.com (T.S.)

**Keywords:** wastewater, enteric viruses, gastrointestinal tract

## Abstract

Human enteric viruses are causative agents in both developed and developing countries of many non-bacterial gastrointestinal tract infections, respiratory tract infections, conjunctivitis, hepatitis and other more serious infections with high morbidity and mortality in immunocompromised individuals such as meningitis, encephalitis and paralysis. Human enteric viruses infect and replicate in the gastrointestinal tract of their hosts and are released in large quantities in the stools of infected individuals. The discharge of inadequately treated sewage effluents is the most common source of enteric viral pathogens in aquatic environments. Due to the lack of correlation between the inactivation rates of bacterial indicators and viral pathogens, human adenoviruses have been proposed as a suitable index for the effective indication of viral contaminants in aquatic environments. This paper reviews the major genera of pathogenic human enteric viruses, their pathogenicity and epidemiology, as well as the role of wastewater effluents in their transmission.

## 1. Introduction

Human enteric viruses are obligate parasites of man that infect and replicate in the gastrointestinal tract of their hosts. Patients suffering from viral gastroenteritis or viral hepatitis may excrete about 10^5^ to 10^11^ virus particles per gram of stool [1], comprising various genera such as adenoviruses, astroviruses, noroviruses, Hepatitis E virus, parvoviruses, enteroviruses (Coxsackie viruses, echoviruses and polioviruses), Hepatitis A virus, and the rotaviruses [2]. Consequently virus concentrations in raw water receiving fecal matter are often high; although viruses cannot reproduce in water they are still capable of causing diseases when ingested, even at low doses [3].

Human enteric viruses are causative agents of many non-bacterial gastrointestinal tract infections, respiratory infections, conjunctivitis, hepatitis and other serious infections such as meningitis, encephalitis and paralysis. These are common in immunocompromised individuals with high morbidity and mortality attributable to these infections in both developed and developing countries. Most cases of enteric virus infections have particularly been observed to originate from contaminated drinking water sources, recreational waters and foods contaminated by sewage and sewage effluents waters [4].

Wastewater treatment processes such as the activated sludge process, oxidation ponds, activated carbon treatment, filtration, and lime coagulation and chlorination only eliminate between 50% and 90% of viruses present in wastewater [5], allowing for a significant viral load to be released in effluent discharge. Due to their stability and persistence, enteric viruses subsequently become pollutants in environmental waters resulting in human exposure through pollution of drinking water sources and recreational waters, as well as foods. The performance of wastewater treatment systems is at present monitored largely by the use of bacterial indicator organisms. Considering that infectious viruses have been isolated from aquatic environments meeting bacterial indicator standards, in some instances in connection with virus related outbreaks [6], the use of bacterial indicators has thus been considered an insufficient tool to monitor wastewater quality because bacterial and viral contaminations are not necessarily associated and linked with each other [7]. This paper reviews the major genera of pathogenic human enteric viruses, their pathogenicities and epidemiology, as well as the role of wastewater effluents in their transmission.

## 2. Major Genera of Human Enteric Viruses: Structure, Pathogenicity and Epidemiology

A diverse range of enteric virus genera and species colonize the gastrointestinal tracts of humans producing a range of clinical manifestations and varying epidemiological features. From a public health perspective, the most important of these are the rotaviruses, adenoviruses, noroviruses, enteroviruses as well as Hepatitis A and E viruses.

### 2.1. Rotaviruses

Rotaviruses are large 70 nm nonenveloped icosahedral viruses that belong to the family Reoviridae [8]. A rotavirus particle consists of a triple-layered protein capsid enclosing 11 segments of a double-stranded RNA genome [9]. The genome encodes six viral proteins (VP1, VP2, VP3, VP4, VP6 and VP7) that make up the viral capsid, and five non-structural proteins (NSP1–NSP5) [10]. The outer capsid is primarily composed of VP4 (a protease-sensitive protein designated P) and VP7 (a glycoprotein designated G) which also forms the basis of defining rotaviruses into P and G serotypes [8]. These two proteins are also determinants of host range. In particular VP4 has been shown to be a determinant of several important functions, such as cell attachment, entry into cells, hemagglutination, and neutralization [9].

There are seven species of rotaviruses, designated A to G, of which groups A–C infect humans [11]. At least 14 G types (G1 to G14) and 20 P types (P [1] to P [20]) have been identified to date, of which 10 G types and five P types have been found in rotaviruses infecting humans [12]. The occurrence of these strains varies spatially and temporally. Type G1P [8] strains are unanimously regarded as the most prevalent and ubiquitous while types G2P [4], G3P [8], and G4P [8] are ubiquitous, but their diffusion is temporal and regional [13].

Rotaviruses infect mature enterocytes in the mid and upper villous epithelium of the host’s small intestines [14]. During the rotavirus replication cycle, virions attach to host cells as triple-layered particles and subsequently enter the cytoplasm by either plasma membrane or endosomal membrane penetration. The attachment of the virus to the cells of the intestinal mucosa is mediated by the structural protein VP4. The infectivity of the virus is enhanced by cleavage of VP4 to produce VP8* and VP5*. The binding of the virus has been proposed to be initially mediated by the cleavage protein VP8* through *N*-acetylneuraminic (sialic) acid residues on the cell surface membrane of the host cell, followed by VP5* or directly by VP5* without the involvement of sialic acid residues. In both cases, the identity of the receptors has remained unclear although, they are thought to be part of lipid micro domains [15]. As a result of cell entry, the outer layer of VP4/VP7 is lost, and the resulting double-layered particles become transcriptionally active, releasing mRNA transcripts through a system of channels that penetrate the middle (VP6) and inner (VP2) capsid layers at each of the icosahedral vertices [16]. After cytosolytic replication in the mature enterocytes of the small intestine, new rotavirus particles can infect distal portions of the small intestine or be excreted in the feces [17].

The pathology of rotavirus infections have been based on a few studies of the jejunal mucosa of infected infants which have revealed shortening and atrophy of villi, distended endoplasmic reticulum, mononuclear cell infiltration, mitochondrial swelling and denudation of microvilli [15]. Rotavirus infection alters the function of the small intestinal epithelium, resulting in the destruction of the mature enterocytes that are responsible for the absorptive function of the villi, while favouring the proliferation of crypt cells that are more secretory resulting in malabsorptive diarrhea [18]. The decreased absorption of Na^+^ ions, results in the transit of undigested mono- and disaccharides, fats, and proteins into the colon. The undigested bolus is osmotically active, resulting in impairment water absorption by the colon which leads to an osmotic diarrhea [18]. The classic presentation of rotaviral infection is fever and vomiting for 2–3 days, followed by non-bloody diarrhea. The diarrhea may be profuse, and 10–20 bowel movements per day are common. When examined, the stool from infected patients is generally devoid of fecal leucocytes [17]. Severe rotavirus gastroenteritis has been associated with pancreatitis [19].

Rotaviruses have been recognized as the leading cause of severe diarrhea in children below 5 years of age, with an estimated 140 million cases and about 800,000 deaths and about 25% of all diarrheal hospital admissions in developing countries each year [20]. Group A rotaviruses are the species most frequently associated with acute gastroenteritis in developed and developing countries. At present there is no available specific treatment for rotavirus infection [21], except prevention through vaccination that has gained licensing in many developed and developing countries [22].

### 2.2. Enteroviruses

Human enteroviruses are members of the family Picornaviridae, which consist of nonenveloped virus particles containing a 7,500-nucleotide single-stranded positive sense RNA genome protected by an icosahedral capsid [23]. The genome encodes four structural proteins, VP1 to VP4 and seven nonstructural proteins implicated in viral replication and maturation. The capsid proteins VP1, VP2, and VP3 are located at the surface of the capsid and are therefore containing epitopes for immunological reaction [23]. There are more than 80 serotypes of human enteroviruses that have been identified on the basis of traditional neutralization tests which aided by the use of molecular based techniques like nucleic acid sequencing has revealed new strains [24]. On the basis of phylogenetic analysis of multiple genome regions, the enterovirus serotypes are classified into four species (Human enterovirus A-D) [25]. These groups consist of 31 serotypes of Echovirus, 23 serotypes of Coxsackie A virus, six serotypes of Coxsackie B virus, three serotypes of Poliovirus and the numbered serotypes of enterovirus [26].

The pathogenicity of enteroviruses is mediated by an arginine-glycine-aspartic acid (RGD) motif found on the viral capsid proteins of the picornavirus family [27]. About seven distinct receptors for different enteroviruses have been identified from human cells, namely; the poliovirus receptor (PVR; CD155), three integrins (*α*2*β*1, *α*v*β*3, and *α*v*β*6), decay-accelerating factor (DAF; CD55), the coxsackievirus-adenovirus receptor (CAR), and intracellular adhesion molecule 1 (ICAM-1) [26]. Typically, the primary site of infection is the epithelial cells of the respiratory or gastrointestinal tract. From the primary infection site, the viruses may spread to secondary sites particularly following viremia. Secondary infection of the central nervous system results in aseptic meningitis or, rarely, encephalitis or paralysis [26].

Most enterovirus infections are asymptomatic or result in only mild illnesses, such as non-specific febrile illness or mild upper respiratory tract infections. However, enteroviruses can also cause a wide variety of clinical illnesses including acute haemorrhagic conjunctivitis, aseptic meningitis, undifferentiated rash, acute flaccid paralysis, myocarditis and neonatal sepsis-like disease [28]. Enteroviruses are the most common etiological agents of human viral myocarditis and are associated with some cases of dilated cardiomyopathy (DCM), which alone afflicts approximately five to eight persons per 100,000 per year worldwide [29]. Enteroviruses are cytopathic, most infections result in tissue specific cell destruction, although some disease manifestations can be a result of host immune response [26].

One of the most distinctive enterovirus diseases is poliomyelitis. It is almost invariably caused by one of the three poliovirus serotypes. Polioviruses may also cause aseptic meningitis or nonspecific minor illness [30]. The normal route of poliovirus infection in naturally permissive hosts begins with infection of the enteric system through oral ingestion of the virus [31]. The cell receptor for all three poliovirus serotypes is CD155, a glycoprotein that is a member of the immunoglobulin super family of proteins [32]. Viral particles initially replicate in the gastrointestinal system, but replication at this site does not result in any detectable pathology [31]. From the primary sites of multiplication in the mucosa, the virus drains into cervical and mesenteric lymph nodes and then to the blood, causing a transient viremia. Most natural infections of humans end at this stage with a minor disease comprising nonspecific symptoms such as sore throat, fever, and malaise. Replication at extraneural sites is believed to maintain viremia beyond the first stage and increase the likelihood of virus entry into the central nervous system. Such extraneural sites might include brown fat, reticuloendothelial tissues, and muscle [32]. As viremia spreads, the infection of dendritic cells and macrophages can aid the transport of the viruses across the blood-brain barrier or transport along neural pathways to infect brain cells [31].

### 2.3. Adenoviruses

Adenoviruses are nonenveloped viruses, about 90 nm in diameter with a linear, double-stranded DNA genome of 34–48 kb and an icosahedral capsid [33]. On the basis of hemagglutination properties as well as DNA sequence homology, tissue tropism, fiber protein characteristics, and other biological properties, human adenoviruses are classified into six species designated A to F [34]. The six species consist of 51 serotypes, defined mainly by neutralization criteria [10]. The virus capsid contains at least nine proteins, of which the hexon, penton base and the fibre proteins are the major capsid proteins [33]. The penton base and the elongated fiber protein form a complex at the vertex of the virus capsid [35].

Adenovirus infection of host epithelial (gastrointestinal and respiratory) cells is mediated by the fibre and penton base capsid proteins. In the case of adenovirus subgroups A and C–F, the attachment to cells is mediated by a high affinity binding of the fiber protein to a 46 kDa membrane protein known as the coxsackie adenovirus receptor (CAR), a member of the immunoglobulin receptor super family serving as a cell to cell adhesion molecule in tight junctions [36]. Subgroup B serotypes such as Ad3, Ad11, and Ad33, as well as the subgroup D serotype Ad37 utilize other receptors such as CD46 and sialic acid [36]. The entry and internalization of the virus into host cells is facilitated by the penton base through the binding of the conserved arginine-glutamine-aspartic acid (RGD) motif to α_v_β_3_ or α_v_β_5_ integrins leading to endocytosis [35].

The major receptor for adenoviruses, CAR is not normally accessible from the apical surfaces. As a result, the initial adenovirus infection is presumed to occur through transient breaks in the epithelium allowing the luminal virus to reach its receptor or during the repair of injured epithelium when CAR might be accessible [37]. Following viral replication, infected cells release viral particles which then filter through the leaky paracellular pathway to emerge on the apical surface where they can spread to other sites of infected tissues [38]. The adenovirus fiber-CAR interactions are also thought to play a role in systemic spread of the virus through the disruption of CAR-mediated endothelial cell-cell adhesion which could facilitate spread to the bloodstream, and virus transport to other sites in the body [38]. The viral infection of the respiratory or gastrointestinal tract may lead to widespread dissemination which can result in diseases such as pharyngitis, conjunctivitis, pneumonia, haemorrhagic cystitis, colitis, hepatitis, or encephalitis which may be fatal in children and immunocompromised patients [39].

Adenovirus infections occur worldwide throughout the year [40]. The serotypes most frequently associated with respiratory infection are members of the subgroup B (Ad3, Ad7, and Ad21), species C (Ad1, Ad2, Ad5 and Ad6) and species E (Ad4) [34].

### 2.4. Noroviruses

Noroviruses are members of the family Caliciviridae [2]. Noroviruses contain a single-stranded positive sense RNA genome of approximately 7.7 kb which is organized into three open reading frames (ORFs). ORF1 encoding a 200-kDa polyprotein that is processed into at least six nonstructural proteins; ORF2 encodes a 60-kDa capsid protein VP1 and ORF3 encoding a basic minor structural protein VP2 [41]. The exterior surface of the virion is composed of a single major protein VP1 that forms the capsid and appears as 32 cup-shaped depressions on the surface showing an icosahedral symmetry on microscopy [42].

The VP1 subunit consists of a shell (S) and a protruding (P) domain that is made up of a middle P1 and a distal P2 subdomains [43]. While the S domain is responsible for the icosahedral shell structure, the P1 and P2 subdomains have been implicated in antigenicity and cellular receptor binding of these viruses [41]. Binding of the VP1 proteins occurs through human histoblood group antigens (HBGAs) as receptors. Human HBGAs are present on the surfaces of red blood cells and more importantly, on the mucosal epithelium [44].

Noroviruses are a major cause of acute viral gastroenteritis, affecting people of all age groups worldwide [45]. Outbreaks of norovirus gastroenteritis can be seasonal or sporadic cases that occur through out the year [46] especially in semiclosed communities such as families, schools, elderly people's homes, hospitals, hotels, and cruise ships [47].

## 3. The Wastewater Treatment Process and Pollution from Viral Pathogens

Municipal wastewater is a mixture of human excreta (sewage), suspended solids, debris and a variety of chemicals that originate from residential, commercial and industrial activities [48]. Raw sewage is a major carrier of disease causing agents, particularly enteric pathogens [1]. The safe treatment of sewage is thus crucial to the health of any community. In subjecting municipal wastewater to treatment before discharge to the environment, the goal is to remove pollutants, both chemical and biological, from the water in order to decrease the possibility of detrimental impacts on humans and the rest of the ecosystem [49]. In the conventional municipal wastewater treatment systems, physical processes such as sedimentation, activated sludge and trickling filters are often used in the decontamination of the wastewater. Human enteric viruses exist in waters as either free-floating or adsorbed onto solid particles. Physical removal of particles by processes like coagulation, flocculation, sedimentation and filtration aids the removal of viruses in wastewater effluents [50]. While these processes remove some viruses associated with large particles, smaller colloidal particles (<10 μm) may pass through these processes to the disinfection stages where they continue to enmesh and protect viruses against disinfectant action [51]. These physical processes remove about 90–99% of the viral load of the wastewater [52]. Additional removal of biological pollutants is achieved by disinfection which often uses chlorine and sometimes ozone, paracetic acid and UV irradiation [53]. Although the combination of all these processes may remove a substantial load of viruses, their efficiencies may vary leading to discharge of pathogenic viruses in the effluents where they subsequently become environmental pollutants [54].

The assessment of the microbiological quality of wastewater effluents has traditionally depended on indicator organisms, such as coliforms or enterococci, which however do not always reflect the risk of other microbial pathogens such as viruses, stressed bacterial pathogens and protozoa [55]. In particular the indicator bacteria survival in water does not correlate with that of enteric viruses [56]. Our recent studies [57–59] have shown that the wastewater treatment facilities in the Eastern Cape Province of South Africa are a veritable source of pathogens in aquatic environments of this study area and negatively impact physico-chemical quality of receiving watershed [60], therefore it is highly probable that they might also be a source of enteric viruses in the aquatic environment.

Viral pathogens have frequently been detected in waters that comply with bacterial standards [61,62]. Human enteric viruses as gastrointestinal tract pathogens are shed in large quantities in the fecal waste of infected individuals and are therefore also found in high quantities in raw sewage [63]. The extent of enteric virus reduction varies according to the sewage treatment system used and the virus type [64].

## 4. Factors Affecting the Removal and Inactivation of Viruses in Wastewater Systems

Enteric viruses in wastewater treatment plants are removed by a combination of irreversible adsorption as well as inactivation by disinfectants [65]. Processes such as coagulation, flocculation, sedimentation and filtration remove viruses adsorbed onto particulate matter [66,67]. The efficiency of removal varies depending on the adsorptive affinities of the virus particles and the adsorbents [68]. Potential adsorbents of viruses in natural waters include sand, pure clays (e.g., montmorillonite, illite, kaolinite, and bentonite), bacterial cells, naturally occurring suspended colloids, and estuarine silts and sediments [50]. Removal rates depend to a great extent on the pH, substrate saturation, redox potential and dissolved oxygen of the system. The protein coats of most viruses gives the viral particles a net charge due to the presence of amino acids such as glutamic acid, aspartic acid, histidine and tyrosine that contain ionized carboxylic and amino groups. Most enteric viruses have a net negative charge at a pH above 5 and a net positive charge below pH 5 [69]. The adsorptive interaction between the virus particle and the adsorbents is a function of isoelectric point of the virus, as well as that of the adsorbent particle and also its hydrophobicity. The variation of dissociation constants among the various polypeptides ensures that most viruses have net charges that vary continuously with varying pH [50]. Adsorption may also be affected by factors such as flow rate and ionic strength. Also flow rate may affect the contact of viruses’ attachment sites, with increasing velocities reducing contact time and therefore the subsequent attachment to sediments. High ionic strength, such as septic tank effluent, favour virus adsorption, with low ionic strength waters, such as rainfall, able to remobilize attached viruses [65].

The inactivation of viruses by disinfection is a process affected by suspended particles. Disinfection relies on the ability of either chemical disinfectant molecules or high-energy photons (in the case of UV disinfection) coming into contact with the viruses [50]. Chemical disinfectants inactivate viruses by either oxidation or disintegration of viral particle, or inhibition of cellular activity [70]. UV disinfection on the other hand relies on the formation of pyrimidine dimers in the DNA/RNA of the target organism, which prevents replication [71]. If contact between the disinfecting agent and the organism is reduced or prevented altogether, then disinfection may be impeded [50]. Organic particles negatively impact the chemical disinfection of viruses by creating a demand for the disinfectant molecules as they penetrate the particle surface. In addition to the disinfectant demand of the particle, particle structure and porosity also plays a role in the shielding of viruses from disinfection [72]. The presence of particle-associated viruses during disinfection of water results in reduced virus inactivation compared to particle-free waters [73].

## 5. Resistance of Enteric Viruses to Disinfectants

The study of the inactivation of enteric viruses following wastewater disinfection is complicated by the low and variable levels of enteric viruses frequently seen in effluents [74]. Research has demonstrated that enteric viruses are inherently more resistant to common disinfectants than bacterial indicators. Tree *et al.* [74], observed that bacterial indicators *Escherichia coli* and *Enterococcus faecalis* were rapidly inactivated by chlorine with inactivation levels of (>5 log_10_ units) while there was poor inactivation (0.2 to 1.0 log_10_ unit) of F^+^-specific RNA (FRNA) bacteriophage (MS2) at doses of 8, 16, and 30 mg/liter of free chlorine. Armon *et al.* [75] also showed that the inactivation levels of naturally occurring coliphages were significantly lower than that of coliforms after chlorination. With regards to UV radiation, enteric adenoviruses have also been shown to be more resistant than bacterial spores [76].

In the United States, the Environmental Protection Agency (EPA) recommends the use of an additional criterion for the evaluation of water disinfection based on viral inactivation. The standard makes use of *Ct* values, defined as disinfectant concentration (*C*) multiplied by the contact time (*t*) between the disinfectant and microorganism. The recommendations direct that public utilities must ensure a 4-log (C*t* _99.99%_) inactivation of viruses [77].

## 6. Consequences of Enteric Virus Persistence in Wastewater Effluents

The inability of wastewater treatment systems to ensure a complete inactivation of viruses in wastewater effluents has serious implications on public health. Virus levels in treated wastewater, measured by cell culture assay, range from 1.0 × 10^−3^ to 1.0 × 10^2^ liter^−1^ depending on the level of treatment [78]. Human enteric viruses can remain stable in the environment for long periods particularly in association with solids in sediments. Goyal *et al.* [79] detected human enteric viruses in sediments obtained from sewage sludge disposal sites in the Atlantic Ocean 17 months after the cessation of sludge dumping. The sediments act as a reservoir from which viruses are resuspended in the water [1]. The persistence of enteric viruses in environmental waters often leads to incidences of human infection through contamination of food, drinking and recreational waters. Enteric viruses have very low infectious doses in the order of tens to hundreds of virions [80]. Even high log reductions in concentration during transport could still result in infectious viruses present in potable water or food [80].

## 7. Water

The discharge of inadequately treated sewage water has a direct impact on the microbiological quality of surface waters and consequently the potable water derived from it. The inherent resistance of enteric viruses to water disinfection processes means that they may likely be present in drinking water exposing consumers to the likelihood of infection. In one study, Human adenoviruses were detected in about 22% of river water samples and about 6% of treated water samples in South Africa [81]. In another study, about 29% of river water samples and 19% of treated drinking water samples in South Africa had detectable levels of enteroviruses [61].

Enteric viruses are the most likely human pathogens to contaminate groundwater. Their extremely small size, allows them to infiltrate soils from contamination sources such as broken sewage pipes and septic tanks, eventually reaching aquifers. Viruses can move considerable distances in the subsurface environment with penetration as great as 67 m and horizontal migration as far as 408 m [80]. In a study in the United States, 72% of groundwater sites were positive for human enteric viruses [82]. In America, the U.S. Environmental Protection Agency (EPA) has proposed a Groundwater Rule that requires public groundwater sites considered to be vulnerable to fecal pollution to be monitored monthly for fecal indicators and that where indicators are found, they must either be a removal of pollution sources or disinfection [82]. Groundwater has been implicated as a common transmission route for waterborne infectious disease in the United States with about 80% waterborne outbreaks attributed to drinking contaminated well water. The enteric viruses most frequently associated with outbreaks are noroviruses and hepatitis A virus [80].

Another important human exposure pathway is through recreational waters. Human enteric viruses have frequently been detected in coastal waters receiving treated wastewater effluents. Xagoraraki *et al.* [83] reported human adenovirus concentrations at the level of 10^3^ virus particles·liter^−1^ in recreational beaches in America. Mocé-Llivina *et al.* [84] detected enteroviruses in 55% of samples from beaches in Spain. The occurrence of viruses in coastal waters results in increased risks of infection to swimmers and divers. The risk of ear, eye, gastrointestinal or respiratory infections is more than twice in polluted than unpolluted beaches [81].

Numerous outbreaks of enteric virus associated diarrhea have been linked to the consumption of water contaminated with viruses. Kukkula *et al.* [85], showed a strong epidemiological risk ratio between the consumption of water contaminated with noroviruses and the outbreak of acute gastroenteritis in Finland. Karmakar *et al.* [6] reported a water-borne outbreak of rotavirus gastroenteritis in India.

## 8. Contamination risks of Foods from Wastewaters with Pollutant Enteric Viruses

Viral contaminants may persist on food surfaces or within foods for extended periods [86]. Pre-harvest contamination may occur in agricultural products subjected to irrigation with reclaimed wastewater, crop fertilization with sewage sludge, or fecal pollution of the areas in which food products are obtained. Numerous studies have attributed outbreaks of enteric virus diseases such as acute gastroenteritis and hepatitis A to the consumption of raw vegetables such as salads. Using epidemiologic data in a case controlled study, Grotto *et al.* [87] showed an association between a norovirus outbreak of gastroenteritis at a military camp in Israel and the consumption of vegetable salads 48 hours preceding the outbreak. In Sweden, Le Guyader *et al.* [86] using sequence based molecular fingerprinting also reported that acute gastroenteritis outbreak was a result of consumption of raspberry cakes contaminated with noroviruses.

Post-harvest contamination of raw food may occur as a result of human handling by workers and consumers, contaminated harvesting equipment, transport containers, contaminated aerosols, wash and rinse water or cross contamination during transportation and storage [88]. Recontamination after cooking or processing, and inadequate sanitation has also been associated with outbreaks of enteric virus infections [89]. In an outbreak of acute viral hepatitis A in Italy, Chironna *et al.* [90] using sequence-based molecular fingerprinting identified a point source of the virus outbreak as a food handler working at a local food outlet. A number of studies have also implicated enteric viruses in disease outbreaks involving contaminated foods [91].

Probably one of the most recognized food borne transmission of enteric virus infections is through the consumption of shellfish grown in sewage polluted marine environments. Shellfish, which includes molluscs such as oysters, mussels, cockles, clams and crustaceans such as crabs, shrimps, and prawns [92], are filter-feeders that result in the bio-concentration of environmentally stable, positive-stranded RNA viruses, such as norovirus, hepatitis A virus and enterovirus in their digestive glands and gills [93]. The risk of human exposure to enteric viral pathogen is increased by the fact that shellfish are often consumed raw, or only slightly cooked [94]. The consumption of shellfish growing in aquatic environments impacted by wastewater effluents or untreated sewage has been associated with numerous outbreaks of gastroenteritis caused by noroviruses as well as cases of hepatitis A [95]. Also, Karamoko *et al.* [96] report mussel samples positive for enteroviruses, and strongly suggests a connection between contaminations of foods by wastewater borne enteric viruses since these mussels were harvested from an area close to a domestic wastewater outlet, more so as mussels harvested from an aquaculture were all found not to be positive for enterovirus. In a similar report, a serious food-borne outbreak in China in 1988 [97–100] was attributed to consumption of clams contaminated with hepatitis A virus from a sewage-polluted community near Shanghai. In Israeli, it was demonstrated that communities using wastewater effluents for irrigation have high incidences of infectious hepatitis as compared to other communities [101]. Although there is little data on the role of wastewater effluents in the propagation of food borne viral diseases, there is high probability that this can be a significant mode of contamination and subsequent disease transmission [101].

## 9. Future Directions

Current safety standards for determining food and water quality typically do not specify what level of viruses should be considered acceptable. This is in spite of the fact that viruses are generally more stable than common bacterial indicators in the environment. While there has been a significant amount of research on the impacts of inadequately treated wastewater effluents in developed countries, the same can not be said of developing countries which coincidentally are faced with a huge burden of infectious diseases emanating from pollution of water bodies with wastewater effluent discharges (von Sperling and Chernicharo [102] most of which remains undocumented, unreported and not properly investigated. The major limitation has been the high cost of establishing facilities for the monitoring and surveillance, especially with enteric viruses that requires specialized laboratories and techniques such as tissue culture, electron microscopy and immunological assays. The use of molecular techniques such as PCR which are relatively rapid and specific however may prove useful for the monitoring of enteric viruses in wastewater effluents. This will have significant benefits in identifying potential avenues of transmission of infectious viruses.

The challenge in ensuring safe water with regards to viral pathogens is that the detection of putative indicators of viral pathogens such as bacteriophages does not always correlate with that of other viruses particularly pathogenic enteric viruses [103]. Human adenoviruses have been proposed as a suitable index for the effective indication of viral contaminants of human origin. For one reason, they are prevalent and very stable; for another, they are considered human specific and are not detected in animal wastewaters or slaughterhouse sewage [103]. Adenovirus strains Adv40 and Adv41 have been associated with diarrheal diseases which can be attributed to consumption of fecal contaminated water and food [104]. As of 2,000 in a study carried in Durham, New Hampshire by Chapron *et al.* [105], adenoviruses together with astroviruses were detected in 51.7 and 48.3% of surface water samples respectively [105]. Adenovirus infections were reported to occur worldwide throughout the year [40], suggesting that there are no seasonal variations in the prevalence of these viruses, thus qualifying these viruses as suitable indicators of human viral pathogens in aquatic environments. Furthermore, PCR-based procedures such as applied real-time PCR that show enough sensitivity to detect not only specific serotypes but also a wide diversity of excreted strains have been described [106]. To this point we can not state exclusively the suitable index for the enteric viruses both in wastewater and drinking waters because there are other proposed indices like Torque teno virus (TTVs) [107], polyomavirus JCPyV [106] which show some degree of suitability as indices. With the increasing popularity of molecular detection methods which are relatively fast and specific compared to the traditional methods such as tissue culture, developing countries may find a solution to the problem of infectious viruses in aquatic environments if such techniques could be incorporated into part of regular monitoring programmes to assess the virus levels in wastewater effluents, and this is a subject of intensive investigation in our group. Microbial Source Tracking (MST) is another promising tool that seeks to predict the source of microbial contamination in the environment, more especially the fecal contamination of aquatic environments [97]. The important aspect of this method is to determine whether the source of fecal contamination is of human or animal origin since viruses are often host-specific [97], and that it may help prevent contamination from its source point. As useful a tool this method may be, it could be negatively influenced by factors like the complexity of the environment under study, the number of sources suspected to be implicated in contamination events, funds available to perform studies, and the technical expertise available to produce and analyze the data, more so in developing countries [97].
